# Three-Dimensional Genome Organization and Virulence in Apicomplexan Parasites

**DOI:** 10.1177/2516865719879436

**Published:** 2019-09-29

**Authors:** Todd Lenz, Karine G Le Roch

**Affiliations:** Department of Molecular, Cell and Systems Biology (MCSB), University of California, Riverside, Riverside, CA, USA

**Keywords:** *Plasmodium*, *Toxoplasma*, *Babesia*, malaria, genome organization, Hi-C, virulence, antigenic variation, *var*, *SICAvar*

## Abstract

Mounting evidence supports the idea that epigenetic, and the overall 3-dimensional (3D) architecture of the genome, plays an important role in gene expression for eukaryotic organisms. We recently used Hi-C methodologies to generate and compare the 3D genome of 7 different apicomplexan parasites, including several pathogenic and less pathogenic malaria parasites as well as related human parasites *Babesia microti* and *Toxoplasma gondii*. Our goal was to understand the possible relationship between genome organization, gene expression, and pathogenicity of these infectious agents. Collectively, our results demonstrate that spatial genome organization in most *Plasmodium* species is constrained by the colocalization of virulence genes that are unique in their effect on chromosome folding, indicating a link between genome organization and gene expression in more virulent pathogens.

**Comment on:** Bunnik EM, Venkat A, Shao J, et al. Comparative 3D genome organization in apicomplexan parasites. *Proc Natl Acad Sci U S A*. 2019;116(8):3183-3192. doi:10.1073/pnas.1810815116. PubMed PMID: 30723152. PubMed Central. PMCID: PMC6386730. https://www.ncbi.nlm.nih.gov/pmc/articles/PMC6386730/.

## Apicomplexan Parasites in Infectious Disease

The environmental and economic impact of apicomplexans—a phylum of obligate intracellular eukaryotic parasites—cannot be understated. *Plasmodium*, the causative agent of malaria, was responsible for an estimated 435 000 deaths in 2017 alone. Relative stagnation in the number of incidences of malaria, afflicting 59 out of every 1000 people worldwide per year from 2015 to 2017, further emphasizes the need for investing in malaria research to discover novel pharmacologic interventions.^1^ Other apicomplexans of importance include *Toxoplasma gondii* and *Babesia microti*, the causative agents of toxoplasmosis and human babesiosis, respectively. Approximately, one-third of the worldwide population are estimated to be infected with *T gondii*, which cause prenatal death or stillbirth in 5% of pregnancies when acquired during the first trimester of gestation.^2^ Babesiosis presents malaria-like symptoms, even resulting in fatality in up to 9% of hospitalized patients infected with *B microti*.^3^

Most antimalarials on the market, and those receiving clinical trials, specifically target *Plasmodium* life cycle progression.^4^ Although these antimalarials are somewhat effective, they are short-term solutions because drug resistance phenotypes in parasite population consistently emerge. Furthermore, a long-lasting and fully protective vaccine is yet to be achieved.^5^ A greater understanding of the mechanisms regulating every phase in the *Plasmodium* life cycle progression will aid in creating effective treatments for all forms of the parasite, including drugs to treat stages involved in the transmission of the parasites from human to mosquitoes and mosquitoes to human. To better understand and block the life cycle progression of these apicomplexan parasites, we need to decipher the mechanisms regulating transcription, including epigenetic factors controlling protein families involved in parasite-host interactions, taking into account genes involved in antigenic variation.

Some apicomplexan parasites, such as *Plasmodium* species, have evolved large gene families called *Plasmodium* interspersed repeats (pir) responsible for parasite survival, pathogenesis, and immune evasion.^6^ Two species, *Plasmodium falciparum* and *Plasmodium knowlesi*, have evolved an additional family of genes—*var* and *SICAvar*, respectively—that provide a means of antigenic variation.^7,8^ They have the ability to adapt their antigenic profile and escape the immune response through changes in their gene expression. In *P falciparum*, there are approximately 60 *var* genes present in the parasite genome but only 1 *var* gene is expressed at any given time.^9^ Over the past few years, extensive research by several teams has been conducted to explore the mechanism of *var* gene regulation *in vitro*. Results have demonstrated that the expression of these genes is most likely regulated by epigenetics and chromatin structure features. Silent *var* genes are marked by H3K9me3 and PfHP1 and are localized to repressed regions of the genome at the periphery of the nucleus.^10-13^ Disruption of histone-modifying enzymes such as histone deacetylase and histone methyltransferases is critical to maintain the regulation of monoallelic var gene expression.^14-18^

**Figure 1.**
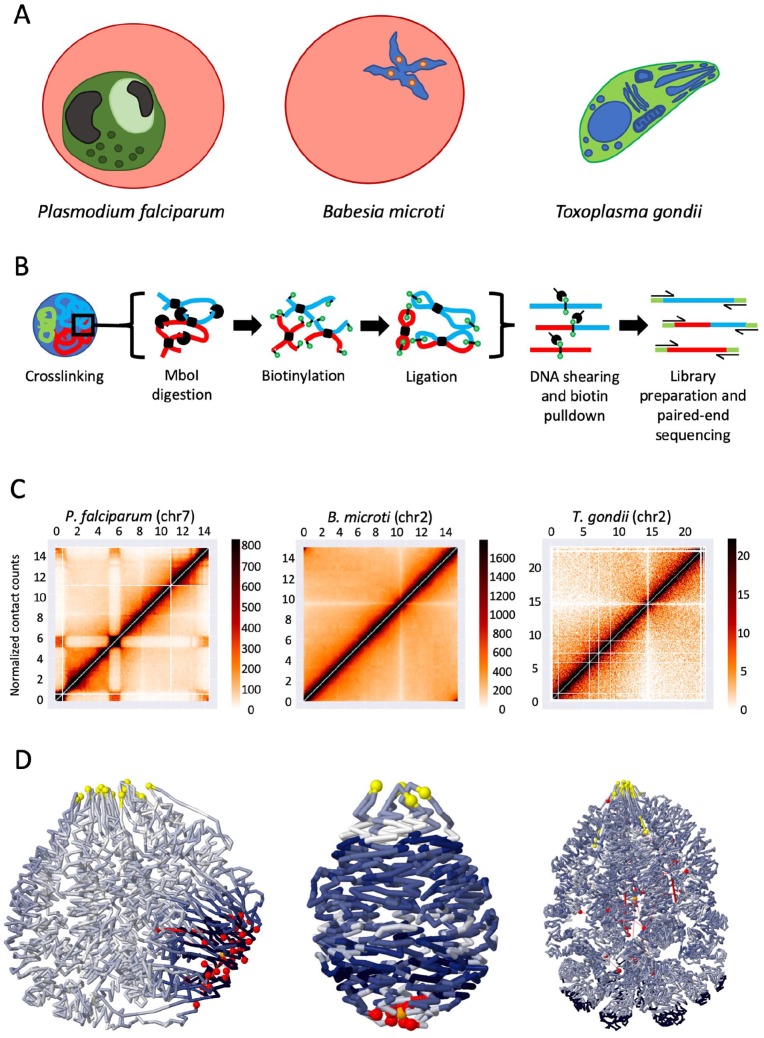
Experimental workflow of sampled species with a corresponding intrachromosomal heatmap and 3D genome model: (A) simplified models of 3 species studied in the experiment; (B) overview of the in situ Hi-C protocol; (C) intrachromosomal contact count heatmaps corresponding to species shown in (A), with domain-like structure shown in *P. falciparum* chr7 due to internal *var* genes; and (D) PASTIS 3D models of entire genomes corresponding to species shown in (A), using a gradient of dark blue to white to represent areas of low to high gene expression, respectively. Yellow spheres represent centromeres, and telomeres are shown as red spheres.

Epigenetic control of transcription is not a unique phenomenon in *Apicomplexans*, as studies have shown similar mechanisms in other single-cell eukaryotes^19^ and complex higher eukaryotes.^20^ Most recently, the spatial organization of chromosomes in eukaryotes has also been extensively studied, and we increasingly understand the role that genome organization plays in mediating gene expression. Due to size constraints of the nuclei in unicellular eukaryotes, processes controlling gene expression vary from those seen in higher multicellular eukaryotes. Much of our current understanding of the systems controlling unicellular epigenetics was obtained studying *Saccharomyces cerevisiae*. However, availability of information regarding other unicellular organisms was limited. Our most recent work investigating genome organization in 7 apicomplexan parasites addresses this knowledge gap. Using chromosome conformation capture (3C) coupled with next-generation sequencing technologies (Hi-C), we examined the role of 3-dimensional (3D) chromatin structure in gene expression and pathogenicity in 5 *Plasmodium* spp.—including the human malaria parasites *P falciparum* and *Plasmodium vivax*, the primate malaria parasite *P knowlesi*, the rodent malaria parasites *Plasmodium berghei* and *Plasmodium yoelii*, and 2 related apicomplexans *T gondii* and *B microti* (Figure 1A).

## Apicomplexan 3D Genome Profiling

Hi-C, which adapts the traditional 3C technique to allow researchers to produce a snapshot of chromatin interactions across an entire genome,^20^ has provided increasing support for the role of epigenetic and chromatin structure in controlling, at least partially, transcription in eukaryotes.^21^ Our group adapted in situ Hi-C methodology^22^ to generate Hi-C libraries in 7 apicomplexan parasites to compare similarities and discrepancies in the genome architecture between the most pathogenetic and less pathogenetic parasitic strains (Figure 1B). We used data generated from 3 distinct *P falciparum* blood stage parasites responsible for the symptoms associated with the disease as well as 2 stages essential for the transmission of the infection, the sexually mature gametocytes and the sporozoites that infect liver cells in human. We also compared data obtained for *P knowlesi*, a primate malaria parasite commonly found in Southeast Asia but that can also infect humans, as well as 2 malaria mouse models, *P yoelii* and *P berghei*. Two additional apicomplexans, *T gondii*, the replicating tachyzoite and the dormant bradyzoite stages, as well as late erythrocytic stage (tetrads) *B microti*, were also included in our study to identify structures or processes unique to the *Plasmodium* genus.

HiC-Pro^23^ was used to analyze approximately 300 million unique read pairs, and chromosomal contacts were collected in contact count matrices at 10-kb resolution (Figure 1C). Following normalization, a consensus 3D genome structure was modeled using the PASTIS 3D modeling toolbox (Figure 1D).^24^ Although analysis of the data and assembly of the 3D structures were error-free for most of the organisms analyzed, several issues arose with the *T gondii* model that could not be remedied given our current understanding of the *Toxoplasma* genome. Prior to this study, the genome assembly for *T gondii* consisted of 14 chromosomes. However, chromosome VIIb and chromosome VIII contain a significantly higher number of interchromosomal interactions than between any other 2 chromosomes. Following the combination with chromosome VIII, we see normalized contacts consistent with the remaining data set. Given the fact that the centromere of chromosome VIIb has not been identified by previous studies, we proposed that both are part of a single chromosome. Once the genome assemblies were fully corrected, we were able to embark on a comparative 3D genome organization study among the apicomplexan parasites investigated.

## Genome Organization in Apicomplexans

We first confirmed that although the genomes of *P falciparum* and *S cerevisiae* are similar in size, the *P falciparum* genome has a more elaborate 3D structure exhibiting domain-like patterns that can be explained by the colocalization of genes involved in antigenic variation.^25^ These results were confirmed by significant interactions observed between *SICAvar* genes in *P knowlesi*. Similarly, we observed some interactions in *P vivax* chr5 between a large locus containing genes encoding for proteins involved in host cell invasion and subtelomeric regions. We tend to speculate that the clustered organization of virulence genes in those *Plasmodium* species allows for increased rates of recombination to generate additional diversity and to orchestrate mutually exclusive gene expression. We further demonstrated that while simpler, the organization of other *Plasmodium* genomes was also most largely driven by their virulence genes. However, as rodent malaria species harbor virulence genes only in subtelomeric regions, their genome organization was unelaborate and similar to fission and budding yeast with clustering of pericentromeric and subtelomeric regions driving the whole 3D structure.^26^

The described features of *Plasmodium* genome organization contrast sharply with the analysis of *B microti* and *T gondii* genomes. The *B microti* genome showed a classical Rabl organization similar to yeast with colocalization of pericentromeric and subtelomeric regions without a clear colocalization of its virulence factors. It is therefore probable that the genome organization in *B microti* is not strongly linked to the regulation of virulence genes as observed in *Plasmodium* parasites. This was further supported by the lack of correlation observed between genome organization and gene expression in this parasite.

Similar results were observed in *T gondii* where virulence genes did not show any colocalization in the nucleus. The only interesting features that we could detect in *T gondii* were the strength of the chromosome territories, similar to what is observed in higher eukaryotes, and the clustering of repressed genes in perinuclear heterochromatin loci. The discrete organization features of the chromatin structure observed between *T gondii* and *Plasmodium* species may be related to the larger size of the *T gondii* genome, its increased number of parasite-specific ApiAP2 transcription factors (67 versus ~27 for *Plasmodium* species),^27^ and its capability of infecting a large number of distinct host cells. It is therefore probable that *Plasmodium* species have evolved into highly specialized organisms that control their virulence factors through a highly restricted mechanism, whereas *T gondii* has retained more properties of their free-living ancestor to regulate its gene expression.

The differences observed between the 3D genome organization and virulence gene expression in the primate and rodent malaria parasites are supported by the absence of several proteins involved in epigenetics features in the genome of rodent malaria parasites, such as the histone methyltransferase PfSET2, that have been demonstrated to be critical to monoallelic *var* gene expression in *P falciparum*.^20^ It is highly possible that the various parasites have evolved different strategies to survive in their respective hosts. One can envision that parasites infecting long-lived primates may require more complex gene regulation patterns to escape the adaptive immune responses and establish long-term infections to ensure transmission to a susceptible host.

Altogether, the comparison of 3D genomes in apicomplexan parasite shows interesting common features and discrepancies in the spatial genome organization of these organisms. Results suggest that genome organization and gene regulation are driven by species-specific mechanisms in this particular phylum. Identifying parasite-specific molecular components controlling chromatin structure may assist the design of novel therapeutic strategies.
